# Domain‐general and domain‐specific functional networks of Broca's area underlying language processing

**DOI:** 10.1002/brb3.3046

**Published:** 2023-05-03

**Authors:** Talat Bulut

**Affiliations:** ^1^ Neurobiology of Language Department Max Planck Institute for Psycholinguistics Nijmegen The Netherlands; ^2^ Department of Speech and Language Therapy Istanbul Medipol University Istanbul Turkey

**Keywords:** Broca's area, domain‐general, domain‐specific, functional connectivity, language, meta‐analytic connectivity modeling

## Abstract

**Introduction:**

Despite abundant research on the role of Broca's area in language processing, there is still no consensus on language specificity of this region and its connectivity network.

**Methods:**

The present study employed the meta‐analytic connectivity modeling procedure to identify and compare domain‐specific (language‐specific) and domain‐general (shared between language and other domains) functional connectivity patterns of three subdivisions within the broadly defined Broca's area: pars opercularis (IFGop), pars triangularis (IFGtri), and pars orbitalis (IFGorb) of the left inferior frontal gyrus.

**Results:**

The findings revealed a left‐lateralized frontotemporal network for all regions of interest underlying domain‐specific linguistic functions. The domain‐general network, however, spanned frontoparietal regions that overlap with the multiple‐demand network and subcortical regions spanning the thalamus and the basal ganglia.

**Conclusions:**

The findings suggest that language specificity of Broca's area emerges within a left‐lateralized frontotemporal network, and that domain‐general resources are garnered from frontoparietal and subcortical networks when required by task demands.

## Graphical abstract

•Domain‐general and ‐specific connectivity of Broca's area was investigated using meta‐analytic connectivity modeling • Language‐specificity of Broca's area arises from a left‐lateralized frontotemporal network • Domain‐general resources are garnered from frontoparietal and subcortical networks when required by task demands

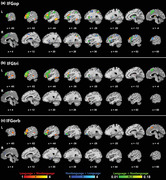

## 1 INTRODUCTION

Broca's area within the left inferior frontal gyrus (IFG), consisting of pars opercularis (IFGop) and pars triangularis (IFGtri) (Amunts et al., 1999), and sometimes extended as Broca's complex to also include pars orbitalis (IFGorb) (Hagoort, 2005a; Xiang et al., 2010), has long been associated with linguistic functions. Among the linguistic functions attributed to this region are grammatical processing involving syntax (Santi & Grodzinsky, 2007) and inflectional morphology (Bulut, 2022b; Laine et al., 1999; Tyler et al., 2005), lexical and compositional semantics (Dapretto & Bookheimer, 1999; Müller et al., 2003; Zhu et al., 2019), and phonological processing (Heim et al., 2003; Matsuo et al., 2010). Recently, distinct linguistic functions have been attributed to different subdivisions of Broca's area. Specifically, IFGop has been associated with syntactic functions, whereas IFGtri and sometimes IFGorb have been involved in semantic functions (Dapretto & Bookheimer, 1999; Goucha & Friederici, 2015; Hagoort & Indefrey, 2014; Newman et al., 2003; Schell et al., 2017; Zaccarella et al., 2017).

Despite abundant research and theoretical claims linking the left IFG with linguistic functions, it is highly controversial whether and to what extent this association is domain‐specific, that is, specific to the language domain, or domain‐general, that is, shared across cognitive domains (Campbell & Tyler, 2018; Fadiga et al., 2009; Fedorenko et al., 2011; Matchin, 2018). In this regard, domain‐general functions attributed to the left IFG, or its subparts, include emotional processing (Belyk et al., 2017; Guha et al., 2020), mathematical and number processing (Hung et al., 2015; Maruyama et al., 2012), action processing (Clos et al., 2013; Papitto et al., 2020), working memory (Chein et al., 2002; Clos et al., 2013; Makuuchi et al., 2009), cognitive control (Clos et al., 2013; Novick et al., 2005, 2010), and music (Asaridou & McQueen, 2013; Heard & Lee, 2020; Koelsch, 2006; Maess et al., 2001), among others. These findings show that the left IFG is recruited for nonlinguistic functions, as well. Given that brain regions assume their domain‐specific roles in the context of a domain‐specific connectivity network, domain‐specific contributions of Broca's area to language processing should be conceptualized within the framework of its domain‐specific connectivity (Friederici, 2011; Hagoort, 2013).

Studies of the functional, effective, and structural connectivity of Broca's area, generally employing resting‐state fMRI, task‐based fMRI coupled usually with dynamic causal modeling, and diffusion tensor imaging, respectively, have provided insights into the connectivity of Broca's area. Specifically, resting‐state fMRI studies identified a largely left‐lateralized functional connectivity pattern for Broca's area involving frontal, temporal, and parietal cortices, as well as several subcortical areas (e.g., the basal ganglia) (Tomasi & Volkow, 2012; Xiang et al., 2010). Structural connectivity research identified various white matter pathways including the superior longitudinal fasciculus (arcuate fasciculus), middle longitudinal fasciculus, inferior fronto‐occipital fasciculus, extreme capsule, external capsule, and uncinate fasciculus that connect Broca's area with the superior and middle temporal gyri as well as with the inferior parietal lobe (supramarginal and angular gyri) (Axer et al., 2013; Glasser & Rilling, 2008; Kellmeyer et al., 2013; Parker et al., 2005; Powell et al., 2006; Saur et al., 2010). Studies of the effective connectivity of Broca's area also delineated the functional connectivity profile of this region both during resting state (Gao et al., 2020), and during various tasks including inhibitory control (Guha et al., 2020), speech production (Eickhoff et al., 2009a), and language processing (den Ouden et al., 2012; Schmithorst et al., 2007; Sonty et al., 2007), highlighting the causal associations between Broca's area and various cortical and subcortical regions. Importantly, this body of research underscored the connection between Broca's area and the posterior superior temporal cortex (Wernicke's area), providing convergent evidence, together with structural connectivity findings, for the primary role of this loop for language processing (den Ouden et al., 2012; Schmithorst et al., 2007; Sonty et al., 2007). Despite progress in understanding the functional network of Broca's area facilitated by this line of research, these techniques are not without limitations. First, resting‐state fMRI and structural connectivity studies reveal task‐independent connectivity patterns of a given brain area, preventing the identification of domain‐specific connectivity networks. Second, although effective connectivity can identify information flow within a functional network in a task‐dependent manner, it is typically utilized in studies with a limited number of participants engaged in a specific task, limiting generalizability of the findings.

A recently developed technique in neuroimaging research that can circumvent these limitations is meta‐analytic connectivity modeling (MACM) (Robinson et al., 2010). MACM combined with activation likelihood estimation (ALE) can be used to identify functional connectivity of a given brain region by calculating its co‐activation patterns using a database of neuroimaging experiments (BrainMap). Importantly, thanks to a detailed taxonomy of experiments enabling searching through the metadata of experiments including behavioral domains, categories, and subcategories (Fox et al., 2005; Lancaster et al., 2012), BrainMap allows estimation of task‐independent, or domain‐general, (Erickson et al., 2017; Robinson et al., 2010), as well as task‐dependent, or domain‐specific, functional connectivity of a brain region (Ardila et al., 2016; Bernal et al., 2015; Viñas‐Guasch & Wu, 2017). Given that large databases of experiments with various tasks and designs are utilized, MACM can produce highly generalizable findings (Samartsidis et al., 2020). Recent MACM investigations of IFG revealed a language network spanning largely left‐lateralized frontal, temporal, and parietal regions, as well as several subcortical structures (Bernal et al., 2015; Bulut, 2022a). Furthermore, striking differences in the language‐related functional connectivity patterns were observed among IFG subdivisions, with the left IFGop co‐activating with a broad network of cortical, subcortical, and cerebellar structures (Bulut, 2022a).

Although MACM has been employed to identify functional connectivity of the left IFGop for language tasks (Bernal et al., 2015) and to parcellate distinct clusters within the left IFGop and identify their connectivity for different functional domains including language (Clos et al., 2013), no previous meta‐analytic connectivity study directly compared functional connectivity of subdivisions of the broadly defined Broca's area, including the left IFGop, IFGtri, and IFGorb, for language and nonlanguage tasks. Although a recent study investigated language‐related functional connectivity of bilateral IFGop, IFGtri, and IFGorb using MACM (Bulut, 2022a), these connectivity patterns were not compared with the connectivity patterns of the relevant regions for other, nonlanguage domains. Given that a functional network identified during language tasks may still involve domain‐general processes, such as working memory and cognitive control, directly exploring divergence (through contrast analyses) and convergence (through conjunction analyses) between the functional network identified for language tasks and that identified for nonlanguage tasks may help disentangle the domain‐specific (specific to language) and domain‐general (shared between language and nonlanguage domains) neural circuitry of Broca's area. Against this background, the present study builds on and extends a previous MACM study on the functional connectivity of IFG for language tasks (Bulut, 2022a). Thus, the aim here is to explore language‐related domain‐specific and domain‐general co‐activation patterns of the left IFGop, IFGtri, and IFGorb by utilizing the MACM method and the BrainMap functional neuroimaging database. To my knowledge, this is the first meta‐analytic connectivity study directly investigating language‐related domain‐specific and domain‐general functional connectivity networks of the opercular, triangular, and orbital parts of Broca's area in a broad sense.

## 2 MATERIALS AND METHODS

The MACM procedure employed in the current study involved defining regions of interest (ROIs) within the left IFG, using the ROIs in addition to several criteria to search the BrainMap database for neuroimaging experiments with language tasks and with tasks other than language that report activation in the relevant ROI, and carrying out ALE analyses to reveal co‐activation network of each ROI for language and other tasks and to compute their contrast and convergence.^1^

### 2.1 Regions of interest

**FIGURE 1:**
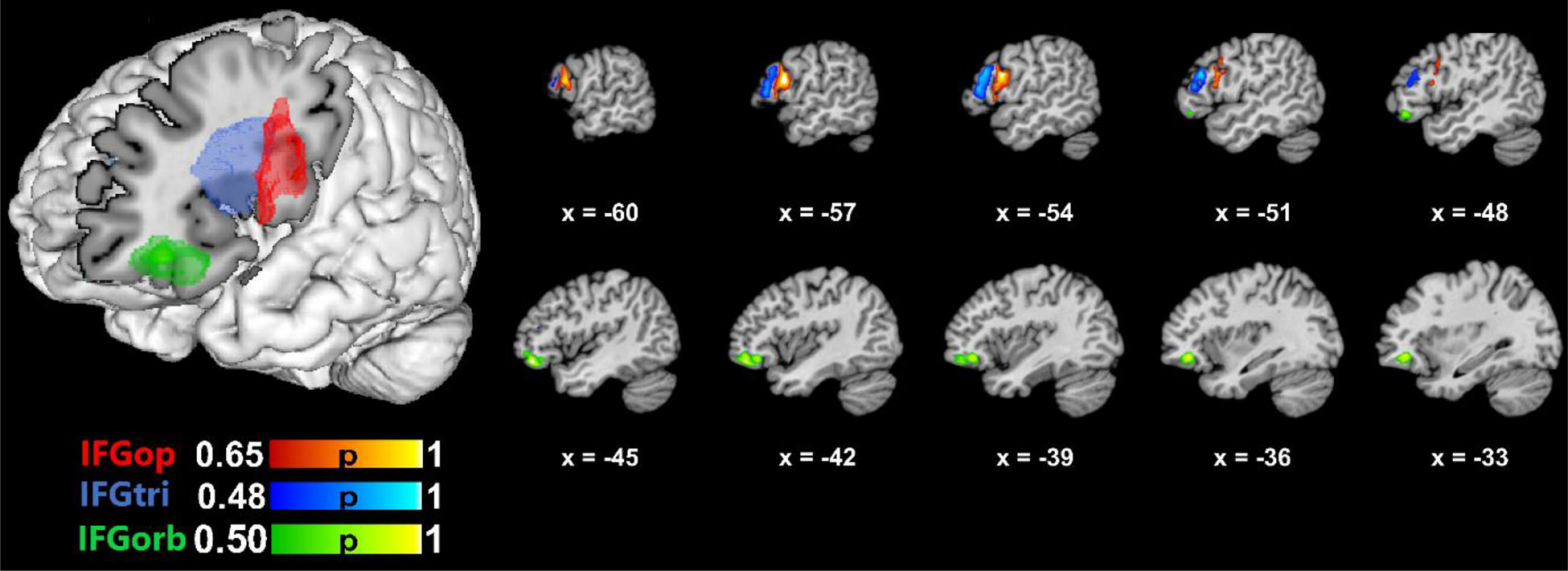
Anatomical 3‐D renderings of the ROIs used in the meta‐analyses. The color bars indicate probability of capturing the relevant anatomical structure within the ROI.

Three ROIs were defined based on the probabilistic, cytoarchitectonic Julich‐Brain atlas (Amunts et al., 2020). As shown in Figure 1, the ROIs corresponded to IFGop, IFGtri, and IFGorb. The ROI maps were obtained from the European Human Brain Project (EHBP) website (https://ebrains.eu), which stores cytoarchitectonic maps for various brain regions (Amunts et al., 2020). The updated versions of the maps currently available were used (v9.2 for IFGop/BA44 and IFGtri/BA45). Given that no separate map is available for IFGorb on the EHBP website, the left Fo6 and Fo7 (v3.2) maps that span parts of the lateral orbitofrontal cortex (Wojtasik et al., 2020) were downloaded from the EHBP website and combined to create an ROI for IFGorb. Bio Image Suite Web (https://bioimagesuiteweb.github.io/webapp/) (Lacadie et al., 2008) was used to ensure that the center of gravity coordinates for Fo6 and Fo7 were within BA47/IFGorb. The Mango software (http://ric.uthscsa.edu/mango/) (Lancaster et al., 2010) was then used to overlay the maps on the MNI template (Colin27_T1_seg_MNI.nii) available on GingerALE's website. Next, the probabilistic maps were thresholded to ensure that each ROI had a probability of representing the relevant brain region greater than 0.48, that the ROIs did not overlap, and that they had similar sizes.^2^ The thresholded maps were used to create the ROIs. To ensure that the intended brain regions were captured, the ROIs were visually inspected using the brain atlases in Mango and using different brain templates in MRIcron (https://www.nitrc.org/projects/mricron) (Rorden & Brett, 2000).

### 2.2 Database search

Database searches were conducted within the BrainMap functional database on 27 September, 2021 using Sleuth Version 3.0.4 (Fox & Lancaster, 2002; Fox et al., 2005; Laird et al., 2005). At the time of the searches, the functional database comprised 3406 papers, 16,901 experiments, 76,016 subjects and, 1,31,598 locations. Two separate searches were conducted: (1) A comprehensive search encompassing all behavioral domains in the database (i.e., action, cognition, emotion, interoception, perception), except speech and language, to capture nonlanguage and nonspeech connectivity patterns of the ROIs (referred to as the nonlanguage search henceforth). (2) A focused search restricted to studies on language recruiting only right‐handed subjects to identify domain‐specific connectivity patterns of the ROIs (referred to as the language search henceforth). Thus, the following search keywords were used in the nonlanguage search: “locations: MNI images of the ROIs,” “experimental context: normal mapping,” “behavioral domain: is not cognition.language, is not action.execution.speech,” “subjects: normal,” “experimental activation: activations only,” “imaging modality: fMRI or PET,” whereas the following search terms were used in the language query: “locations: MNI images of the ROIs,” “experimental context: normal mapping,” “behavioral domain: cognition.language,” “experimental activation: activations only,” “subjects: normal,” “handedness: right,” “imaging modality: fMRI or PET.” Restriction of the searches to “normal mapping” and “normals” ensured that only the experiments conducted with healthy subjects were included. The ROIs defined as explained above were separately included as a search criterion in the database searches. The language search was intended to yield only language‐relevant activations, hence, only the “cognition.language” behavioral domain encompassing all linguistic levels (phonology, orthography, semantics, syntax, speech) was used, but not “action.execution.speech” to exclude action‐related processes of articulation. However, to ensure linguistic processes are excluded from the nonlanguage search as much as possible, both “cognition.language” and “action.execution.speech” were used as exclusion criteria in that search. The same ROIs were used in both the language and nonlanguage searches. The results identified by each database search are summarized in Table 1 below.^3^

**TABLE 1:** Language and nonlanguage search results for each ROI

ROI	Papers	Subjects	Experiments	Locations
Nonlanguage search				
IFGop	170	2695	203	3684
IFGtri	91	1846	99	1571
IFGorb	79	1343	89	1024
Language search				
IFGop	72	1100	95	1394
IFGtri	68	926	88	1264
IFGorb	29	436	34	538

Table 2 summarizes the distribution of experiments identified as a result of language and nonlanguage searches across BrainMap behavioral domains, categories, and subcategories (for details on the BrainMap taxonomy of experiments, please refer to Fox et al., 2005; Lancaster et al., 2012). It should be noted that it is possible for an experiment to relate to more than one behavioral domain, category, and subcategory. As illustrated in Table 2, the nonlanguage search identified experiments in all behavioral domains excluding action.execution.speech and cognition.language, while the language search identified the experiments categorized as cognition.language. Of note, since nonlanguage cognitive domains or categories were not added in the language search as exclusion criteria to ensure as broad coverage of language‐related experiments as possible, a subset of the experiments identified in the language search also related to some other domains (e.g., perception.audition). Take, for example, a picture naming study included in the current meta‐analyses (Wilson et al., 2009). In an overt picture naming paradigm, participants were asked to name pictures while they did nothing in response to scrambled pictures, which were created by randomly shuffling parts of many pictures. From this study, the contrast of *naming pictures > fixation* was identified in the language search for IFGop in the present study, because it was categorized by BrainMap into the language domain (cognition.language.semantics, cognition.language.speech), and because it reported activation in IFGop. However, this contrast was also categorized as perception.vision (unspecified) and cognition.attention, in addition to the language domains, given that the contrast involves processing of visual stimuli, that is, pictures, and requires attention to decide when to execute the naming process (proper pictures vs. scrambled pictures). Another example is a study with a covert reading paradigm involving emotional words compared to baseline conditions (Beauregard et al., 1997). Two contrasts from this study contributed to the language analyses for IFGtri and IFGorb, but they were also categorized within the emotion.negative (unspecified) domain. Importantly, although these experiments contributed to the language meta‐analyses in the current study, they were not included in the nonlanguage analyses as they were also categorized in the language domain (note that being categorized in the cognition.language or action.execution.speech domains is an exclusion criteria for the nonlanguage domain here). This meant that an experiment or contrast could not be in both comparison sets (language and nonlanguage).

**TABLE 2:** Distribution across BrainMap behavioral domains of the experiments entered in the language and nonlanguage MACM analyses for each ROI

Domain	Category (Subcategory)	Nonlanguage search			Language search		
		IFGop	IFGtri	IFGorb	IFGop	IFGtri	IFGorb
Action	Execution (Speech)	0	0	0	2	3	1
Action	Execution (Unspecified)	24	1	3	2	1	0
Action	Imagination	12	3	0	0	0	0
Action	Inhibition	6	3	3	0	0	0
Action	Motor Learning	4	0	0	0	0	0
Action	Observation	6	2	1	2	1	1
Action	Preparation	3	0	0	1	0	0
Action	Rest	0	0	0	0	0	0
Cognition	Attention	37	25	10	3	1	0
Cognition	Language (Orthography)	0	0	0	10	8	4
Cognition	Language (Phonology)	0	0	0	25	8	4
Cognition	Language (Semantics)	0	0	0	37	59	27
Cognition	Language (Speech)	0	0	0	38	30	5
Cognition	Language (Syntax)	0	0	0	10	5	2
Cognition	Language (Unspecified)	0	0	0	6	6	0
Cognition	Memory (Explicit)	11	16	15	2	3	2
Cognition	Memory (Implicit)	0	0	0	0	0	0
Cognition	Memory (Working)	26	5	8	2	0	1
Cognition	Memory (Unspecified)	0	3	3	0	0	0
Cognition	Music	24	5	4	0	1	0
Cognition	Reasoning	16	18	13	4	6	3
Cognition	Social cognition	8	6	7	1	1	0
Cognition	Somatic	1	0	1	0	0	0
Cognition	Spatial	2	0	2	0	0	0
Cognition	Temporal	3	1	0	0	1	0
Emotion	Intensity	1	0	0	0	0	0
Emotion	Negative (Anger)	1	3	3	0	0	0
Emotion	Negative (Anxiety)	1	1	1	0	0	0
Emotion	Negative (Disgust)	2	1	1	0	0	0
Emotion	Negative (Embarrassment)	1	2	0	0	0	0
Emotion	Negative (Fear)	2	5	2	0	0	0
Emotion	Negative (Guilt)	1	1	0	0	0	0
Emotion	Negative (Punishment/loss)	0	0	2	0	0	0
Emotion	Negative (Saddness)	0	2	5	0	0	0
Emotion	Negative (Unspecified)	12	3	7	0	1	1
Emotion	Positive (Humor)	0	0	0	0	0	0
Emotion	Positive (Happiness)	0	3	2	0	0	0
Emotion	Positive (Reward/gain)	9	6	9	0	0	0
Emotion	Positive (Unspecified)	6	3	2	0	0	0
Emotion	Valence	4	2	1	0	0	0
Interoception	Baroregulation	0	0	0	0	0	0
Interoception	Gastrointestinal/genitourinary	2	0	1	0	0	0
Interoception	Heartbeat detection	0	0	0	0	0	0
Interoception	Hunger	1	0	1	0	0	0
Interoception	Osmoregulation	1	0	0	0	0	0
Interoception	Respiration regulation	1	0	0	0	0	0
Interoception	Sexuality	12	1	2	0	0	0
Interoception	Sleep	0	0	1	0	0	0
Interoception	Thermoregulation	1	0	0	0	0	0
Interoception	Thirst	1	0	0	0	0	0
Interoception	Vestibular	1	0	0	0	0	0
Perception	Audition	12	4	6	5	5	3
Perception	Gustation	2	0	7	0	0	0
Perception	Olfaction	0	1	1	0	0	0
Perception	Somesthesis (Pain)	10	3	2	0	0	0
Perception	Somesthesis (Unspecified)	7	2	0	0	0	0
Perception	Vision (Color)	0	1	0	0	0	0
Perception	Vision (Motion)	2	0	3	0	0	0
Perception	Vision (Shape)	3	3	3	1	0	0
Perception	Vision (Unspecified)	14	6	4	2	3	0

The foci identified in each search were grouped by experiment using the most conservative approach (Turkeltaub et al., 2012); that is, foci reported in multiple experiments in a single study were combined and entered into the meta‐analyses as a single experiment to prevent a single experiment from overinfluencing the results. The icbm2tal transform was implemented to automatically convert coordinates reported in Talairach space into MNI space (Laird et al., 2010; Lancaster et al., 2007).

### 2.3 ALE analyses

Convergence of co‐activations for each ROI was computed through ALE analyses using GingerALE 3.0.2 (Eickhoff et al., 2009b; Eickhoff et al., 2012). To that end, ALE analyses were performed using the activation coordinates identified for each ROI as a result of the language and nonlanguage searches. Standard procedures were implemented to carry out the ALE analyses as reported in previous research (Cieslik et al., 2015; Müller et al., 2017; Wojtasik et al., 2020). In particular, 3D Gaussian probability distributions centered at each foci group were generated using a full‐width half‐​maximum, which was calculated based on the sample size in each experiment (Eickhoff et al., 2009b). Then, the union of modeled activation maps was acquired to compute voxel‐wise ALE scores. Afterwards, the union of these activation probabilities was compared against the null hypothesis of random spatial association between the experiments. Finally, the *p*‐value distributions derived from these probabilities were thresholded at a voxel‐level uncorrected cluster‐forming threshold of *p* < .001 and a cluster‐level corrected threshold of *p* < .05 (family‐wise error corrected for multiple comparisons), with 10,000 thresholding permutations.

To compare language and nonlanguage co‐activation patterns for each ROI, bidirectional contrast or subtraction analyses (language > nonlanguage, nonlanguage > language) as well as conjunction analyses (language ∩ nonlanguage) were performed using the Contrast Datasets utility in GingerALE. The network identified by the contrast of language > nonlanguage is interpreted as the domain‐specific (language‐specific) network, while the conjunction of language ∩ nonlanguage is taken to reflect the domain‐general (shared between language and nonlanguage domains) network of the ROIs. The nonlanguage > language network, on the other hand, corresponds to co‐activations of the ROIs specific to nonlanguage domains. In addition to these main analyses which combine foci from all domains other than language and speech into a general nonlanguage set, exploratory analyses were conducted to identify domain‐specific and domain‐general networks of the ROIs within individual behavioral domains. The exploratory analyses were conducted to see, across different behavioral domains, whether the domain‐specific co‐activation patterns would be consistent and whether and how the domain‐general networks would differ. Given that inclusion of at least 17–20 experiments in ALE meta‐analyses has been recommended to obtain enough power for identification of small effect sizes and to ensure that the results are not overly influenced by individual studies (Eickhoff et al., 2016; Müller et al., 2018), the upper bound of this recommendation was adopted. Thus, exploratory analyses were performed for the individual behavioral domains in Table 2 which contributed 20 or more experiments. These are action.execution, cognition.attention, cognition.memory.working, and cognition.music for IFGop, and cognition.attention for IFGtri. It should be noted that the ALE subtraction analysis applies permutation significance testing, which controls for differences in the number of papers on each side of the comparison (Eickhoff et al., 2011; Erickson et al., 2017).

Since GingerALE conducts contrast analyses based on already thresholded single‐dataset images, and since cluster‐level inference is not currently available in GingerALE for contrast analyses (Hoffman & Morcom, 2018), an uncorrected threshold of *p* < .05 with an extent threshold (minimum cluster size) of 100 mm^3^ was used for the contrast and conjunction analyses, as applied in previous research (Bulut, 2022a; D'Astolfo & Rief, 2017; Garrison et al., 2013; Hobeika et al., 2016; Kollndorfer et al., 2013; Papitto et al., 2020). The Talairach Daemon embedded in GingerALE was used to generate anatomical labels as the nearest gray matter within 5 mm for the activation peaks (Lancaster et al., 1997, 2000). The Mango software (Lancaster et al., 2010) was used to visualize the ALE results, which were overlaid on the MNI template (Colin27_T1_seg_MNI.nii) downloaded from the GingerALE website. The Sleuth files (workspace files including metadata of the experiments identified in each search, and text files containing the foci obtained from the identified experiments and entered in the meta‐analyses) as well as the GingerALE output files for each meta‐analysis are available at https://doi.org/10.17632/tfg4pryhf9.1.

### 2.4 Results of main analyses

**FIGURE 2:**
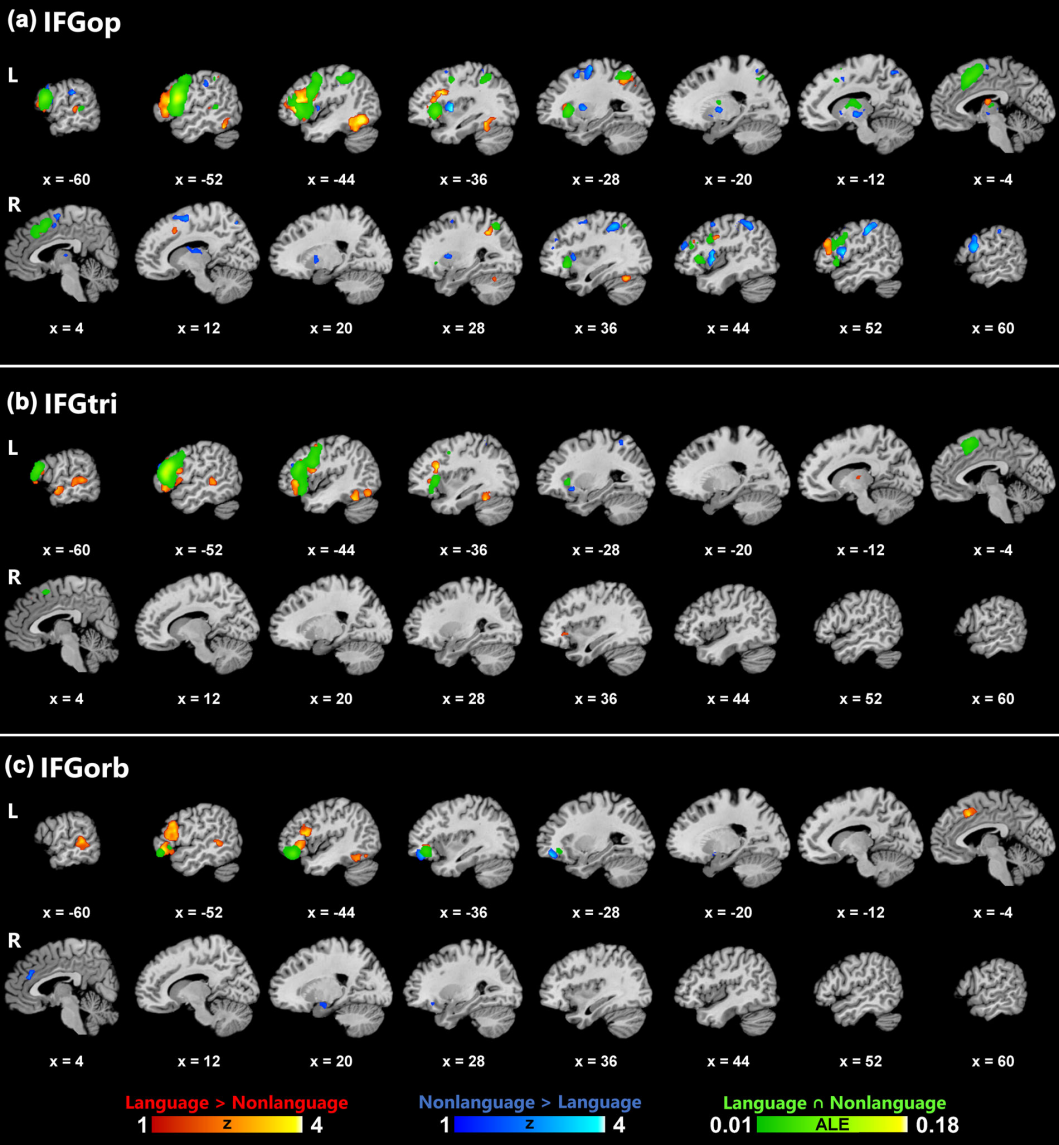
Results of contrast and conjunction analyses on experiments categorized within the language and nonlanguage domains for each ROI. Color bars indicate *Z* scores for contrast and ALE scores for conjunction analyses.

The contrast and conjunction results of the language and nonlanguage ALE analyses for the left IFGop, IFGtri, and IFGorb are summarized in Tables 3 and 4, and illustrated in Figure 2. The most widespread co‐activation pattern for both contrast analyses and the conjunction analysis was observed for IFGop, followed by IFGtri and, lastly, by IFGorb. The domain‐specific (language > nonlanguage) co‐activation network of IFGop was mostly left‐lateralized, spanning left frontal (IFG, MFG, precentral gyrus), temporal (fusiform gyrus, STG) and parietal (SPL, IPL, precuneus) structures, but also involved several right‐hemispheric clusters in the right frontal (MFG, IFG, precentral gyrus) and limbic (cingulate gyrus) lobes. The nonlanguage co‐activation patterns of IFGop, on the other hand, were mainly right‐lateralized, involving right frontal (insula, IFG, MFG, SFG, precentral gyrus, FGmed), limbic (cingulate gyrus), and parietal (IPL, postcentral gyrus, precuneus) structures, but also including left frontal (IFG, MFG, precentral gyrus), parietal (precuneus, IPL, postcentral gyrus), and limbic (cingulate gyrus) regions. The domain‐general network of IFGop revealed by the conjunction analysis of language and nonlanguage co‐activations identified largely left‐lateralized frontal, parietal, and temporal regions as well as several peaks in the right frontal, limbic, and parietal cortices.

**TABLE 3:** Contrast and conjunction results for IFGop

			MNI Coordinates				
Cluster	Anatomical label (Nearest gray matter within 5 mm)	BA	*x*	*Y*	*Z*	*Z*/ALE	Cluster size (mm^3^)
IFGop Language > Nonlanguage						*Z*	
1	L IFG	45	−46.1	25.9	14	3.89	19,512
	L IFG	46	−46	29.5	10.5	3.29	
	L Insula		−38	20	−6	3.04	
	L Claustrum		−34	18	−4	2.97	
	L MFG	10	−46	48	8	2.75	
	L Precentral gyrus	44	−46	14	6	2.68	
	L Insula	13	−30	28	6	2.49	
	L Insula	13	−46	12	0	2.35	
	L Precentral gyrus	44	−60	14	2	2.19	
	L MFG	10	−42	48	16	1.88	
2	L Fusiform gyrus	37	−45.4	−59.5	−14.5	3.89	5960
	L Fusiform gyrus	37	−40.7	−51.3	−17.7	3.54	
	L Tuber (Cerebellum)		−50	−54	−25	3.54	
	L Fusiform gyrus	37	−44	−44	−18	3.35	
	L Temporal lobe (Subgyral)	37	−46	−48	−10	3.06	
3	R MFG	46	54	32	22	3.29	2432
	R IFG	45	46	26	12	3.04	
	R IFG	45	51	27	11	2.76	
4	L SPL	7	−26	−54	44	3.29	1928
	L Precuneus	19	−28	−60	44	3.04	
	L Precuneus	31	−26	−74	32	2.43	
5	R Culmen (Cerebellum)		38	−60	−26	3.72	1104
6	L STG	22	−58	−31	6	2.77	632
7	R Cingulate gyrus	32	14	16	40	2.50	512
	R Cingulate gyrus	32	4	20	40	1.94	
8	L Thalamus		−4	−10	14	2.85	504
9	No gray matter found		28	−52	39	3.54	488
10	R Precentral gyrus	6	44	0	30	3.09	312
	R Precentral gyrus	6	44	−4	32	2.93	
11	L IPL	40	−50	−48	50	2.09	208
IFGop Nonlanguage > Language						Z	
1	R Insula	13	45.5	5.5	4.4	2.86	5360
	R Insula	13	43	5	−4	3.43	
	R IFG	44	59.5	7.5	17.5	3.43	
	R Precentral gyrus	6	60	6	30	2.77	
2	R IPL	40	54.7	−28.7	43.7	3.89	4440
	R Postcentral gyrus	2	54	−28	38	3.72	
	R IPL	40	40.9	−42.9	48.3	3.06	
	R IPL	40	50	−40	54	3.35	
3	L Claustrum		−35.7	−0.6	3	3.89	3552
	L Subthalamic nucleus (Midbrain)		−8	−10	−6	3.09	
	L Lateral globus pallidus		−22	−4	2	2.70	
	L Pulvinar (Thalamus)		−6	−26	2	2.20	
4	L MFG	6	−23.5	−10.5	58	3.72	2312
	L MFG	6	−30	10	52	2.82	
	L MFG	6	−32	8	56	2.64	
5	R FGmed	6	18	6	60	3.54	2256
	R FGmed	6	10	−4	58	3.06	
	R FGmed	6	6	−2	50	2.54	
	R Cingulate gyrus	24	6	0	46	2.35	
6	L IFG	9	−58	10	30	2.67	1656
	L Precentral gyrus	6	−64	6	18	2.59	
7	R Medial dorsal nucleus (Thalamus)		8	−12	10	2.33	1320
	R Caudate body		12	−2	18	2.09	
	R Thalamus		16	−17	12	1.92	
8	R MFG	6	40	6	52	2.69	1112
	R MFG	6	44	6	52	2.64	
	R Precentral gyrus	6	36	−4	54	2.44	
	R MFG	6	50	8	48	2.35	
9	R Putamen		28	4	8	2.86	1072
	R Lateral globus pallidus		18	4	−4	2.48	
10	R SFG	9	46	46	22	2.60	776
11	L Precuneus	7	−14	−60	56	2.76	664
12	L IPL	40	−54	−30	38	2.48	496
	L IPL	40	−48	−26	36	2.28	
13	R Precuneus	7	12	−70	52	3.06	304
	R Precuneus	7	14	−76	54	2.75	
14	L Postcentral gyrus	40	−60	−26	24	3.04	280
	L IPL	40	−60	−28	28	2.88	
15	R Insula	13	32	26	10	1.92	232
	R Insula	13	32	30	0	1.85	
16	L Cingulate gyrus	24	−10	4	44	2.15	232
17	R MFG	9	34	38	30	2.06	152
18	L Caudate head		−10	10	−2	2.24	112
19	L IPL	40	−36	−44	60	1.98	112
20	L Putamen		−18	6	−8	2.00	104
	L Putamen		−18	10	−6	1.88	
IFGop Language ∩ Nonlanguage						ALE	
1	L IFG	44	−54	12	18	0.173	27,752
	L Claustrum		−32	22	0	0.087	
	L IFG	13	−44	26	2	0.064	
	L Precentral gyrus	6	−46	4	48	0.049	
	L MFG	46	−46	36	12	0.043	
	L MFG	46	−48	34	18	0.039	
	L MFG	46	−44	32	20	0.037	
2	L FGmed	32	−4	16	46	0.058	9944
	L FGmed	6	−2	10	50	0.055	
	R Cingulate gyrus	32	8	16	42	0.039	
	R Cingulate gyrus	32	4	24	36	0.034	
	R FGmed	8	6	32	40	0.026	
3	L IPL	40	−44	−42	48	0.047	6224
	L Precuneus	7	−26	−64	46	0.043	
	L IPL	40	−38	−46	42	0.035	
4	R Insula		36	24	−8	0.046	3480
	R IFG	13	42	24	2	0.043	
	R Insula	13	50	16	−4	0.037	
5	R IFG	9	48	8	28	0.039	2600
	R MFG	46	52	22	16	0.032	
	R MFG	9	50	24	26	0.029	
	R MFG	9	46	26	24	0.028	
	R MFG	9	54	22	26	0.025	
6	L Thalamus		−8	−10	10	0.039	1760
	L Putamen		−12	2	6	0.023	
7	R SPL	7	30	−58	46	0.038	1056
8	L STG	22	−54	−40	8	0.030	744
	L MTG	22	−60	−36	4	0.029	
9	R MFG	46	46	32	18	0.030	232
	R MFG	9	46	36	26	0.024	

*Note*: MNI Coordinates correspond to cluster peaks, and anatomical labels indicate gray matter nearest to the cluster peaks. Please refer to the online data repository for cluster analyses with full reports of structures included in each cluster. FGmed, Medial frontal gyrus; IFG, Inferior frontal gyrus; IPL, Inferior parietal lobule; L, Left; MFG, Middle frontal gyrus; MTG, Middle temporal gyrus; R, Right; SFG, Superior frontal gyrus; SPL, Superior parietal lobule; STG, Superior temporal gyrus.

**TABLE 4:** Contrast and conjunction results for IFGtri and IFGorb

Cluster	Anatomical label (Nearest gray matter within 5 mm)	BA	MNI Coordinates			*Z*/ALE	Cluster size (mm^3^)
			*x*	*Y*	*z*		
IFGtri Language > Nonlanguage						*Z*	
1	L MFG	9	−41.5	19.2	21.7	3.89	9832
	L IFG	47	−56	29	−10	3.54	
	L IFG	44	−46	19.5	6	0.00	
	L IFG	45	−48	32	−8	3.16	
	L IFG	47	−46	34	−14	3.12	
	L IFG	44	−56	14	14	2.75	
	L IFG		−58	20	−4	2.62	
	L STG	22	−56	10	−2	2.61	
	L MFG	9	−54	28	32	2.18	
2	L Culmen (Cerebellum)		−42	−50	−26	3.89	3304
	L Culmen (Cerebellum)		−37.3	−49.3	−26	3.72	
	L Declive (Cerebellum)		−42.7	−70.7	−18	2.93	
3	L MTG	22	−58	−38	2	3.12	2472
	L STG	22	−50	−36	2	3.06	
	L MTG	21	−56	−28	−8	2.86	
	L MTG		−58	−32	2	2.56	
4	L STG	22	−57	−7	−8	0.00	1096
5	L Precentral gyrus	6	−46	2	48	2.74	632
6	R IFG	47	38	28	−4	2.44	328
	R IFG	47	38	32	−6	2.37	
7	L Thalamus		−10	−14	6	2.10	296
8	R FGmed	8	8	30	44	2.19	128
9	R SFG	6	9	19	48	0.00	104
IFGtri Nonlanguage > Language						*Z*	
1	L MFG	46	−48	36	22	2.38	720
2	L SPL	7	−32	−50	54	2.45	648
	L Precuneus	7	−28	−54	58	2.24	
	L IPL	7	−32	−56	52	2.23	
3	L Claustrum		−30	16	−12	2.55	312
IFGtri Language ∩ Nonlanguage						ALE	
1	L IFG	45	−52	30	14	0.144	24616
	L MFG	46	−52	28	18	0.140	
	L IFG	9	−44	8	28	0.052	
	L Insula	13	−34	22	0	0.045	
	L IFG	47	−46	22	−8	0.044	
	L Precentral gyrus	6	−44	−2	50	0.030	
2	L SFG	6	−4	16	56	0.049	4080
	L FGmed	32	−6	18	44	0.037	
IFGorb Language > Nonlanguage						*Z*	
1	L IFG	45	−48.5	24.6	4.5	3.19	9208
	L IFG	45	−46	21.3	8	0.00	
	L IFG	9	−48.3	20.3	19.7	2.76	
	L IFG	44	−50	14	12	3.29	
	L IFG	45	−52	24	16	3.24	
	L IFG	45	−54	26	12	3.09	
	L IFG	47	−34	34	−2	2.62	
	L IFG	47	−54	20	−12	2.36	
2	L MTG	21	−64	−40	2	3.72	3032
	L MTG	22	−57	−41	3	3.16	
	L MTG	21	−67	−36	1	3.35	
	L MTG	22	−68	−34	6	3.35	
	L MTG	22	−56	−46	4	3.29	
	L MTG	22	−63	−44	4	1.92	
3	L Fusiform gyrus	37	−48	−58	−18	3.06	1176
	L Fusiform gyrus	37	−48	−66	−17	2.85	
4	L Cingulate gyrus	32	−3.3	16	41.3	3.72	1176
	L FGmed	6	‐4	10	50	2.18	
IFGorb Nonlanguage > Language						Z	
1	L MFG	11	−31.4	45.4	−17.4	3.89	1872
2	R Cingulate gyrus	32	4	34	24	2.54	632
3	R Parahippocampal gyrus	34	22	−12	−18	2.26	512
	R Parahippocampal gyrus	28	18	−6	−16	1.89	
4	R Putamen		22	26	−14	2.33	344
5	L Putamen		−20	4	−14	1.76	144
IFGorb Language ∩ Nonlanguage						ALE	
1	L MFG	47	−46	36	−14	0.075	7232
	L IFG	47	−50	24	−6	0.024	

*Note*: MNI Coordinates correspond to cluster peaks, and anatomical labels indicate gray matter nearest to the cluster peaks. Please refer to the online data repository for cluster analyses with full reports of structures included in each cluster. FGmed, medial frontal gyrus; IFG, inferior frontal gyrus; IPL, inferior parietal lobule; L, left; MFG, middle frontal gyrus; MTG, middle temporal gyrus; R, right; SFG, superior frontal gyrus; SPL, superior parietal lobule; STG, superior temporal gyrus.

The domain‐specific co‐activation patterns of IFGtri and IFGorb were almost exclusively left‐lateralized, and, similar to IFGop, exhibited co‐activation in the left frontal (IFG, MFG) and temporal (fusiform gyrus, STG, and MTG) lobes, but not in the parietal lobe. However, domain‐specific involvement of posterior middle and superior temporal regions showed differences among the ROIs, with IFGop co‐activating with these regions more superiorly than IFGtri and IFGorb. In addition, only IFGtri co‐activated with anterior STG and MTG as part of the domain‐specific network. The nonlanguage co‐activation networks of IFGtri and IFGorb were markedly different from those of IFGop, in which the former were predominantly left‐lateralized. While the nonlanguage co‐activation network of IFGtri included the left frontal (MFG) and parietal (SPL, IPL, precuneus) cortices, that of IFGorb spanned the left frontal (MFG) and the right limbic (cingulate gyrus, parahippocampal gyrus, amygdala) lobes. The domain‐general co‐activation network of IFGtri largely overlapped with that of IFGop in the left superior‐dorsal frontal (IFG, MFG, FGmed, precentral gyrus) regions, but differed from that of IFGorb, which mainly involved the orbitofrontal cortex. Interestingly, the co‐activation patterns of IFGorb demonstrated a lateral‐to‐medial gradient within the left orbitofrontal cortex with gradually shifting lateral, lateral‐medial, and medial patterns for the domain‐specific, domain‐general, and nonlanguage networks, respectively.

As for subcortical and cerebellar co‐activation patterns, only IFGop and IFGtri co‐activated with subcortical structures (left thalamus) as part of their domain‐specific networks. However, all ROIs exhibited some subcortical co‐activation primarily within the putamen and/or thalamus as part of their nonlanguage network. Additionally, IFGop also co‐activated with the bilateral caudate nuclei within its nonlanguage network. Domain‐specific networks of all ROIs showed some co‐activation with the left cerebellum as part of a larger cluster also overlapping with the fusiform gyrus. However, only IFGop exhibited distinct co‐activation with the right cerebellum within its domain‐specific network.

### 2.5 Results of exploratory analyses

**FIGURE 3:**
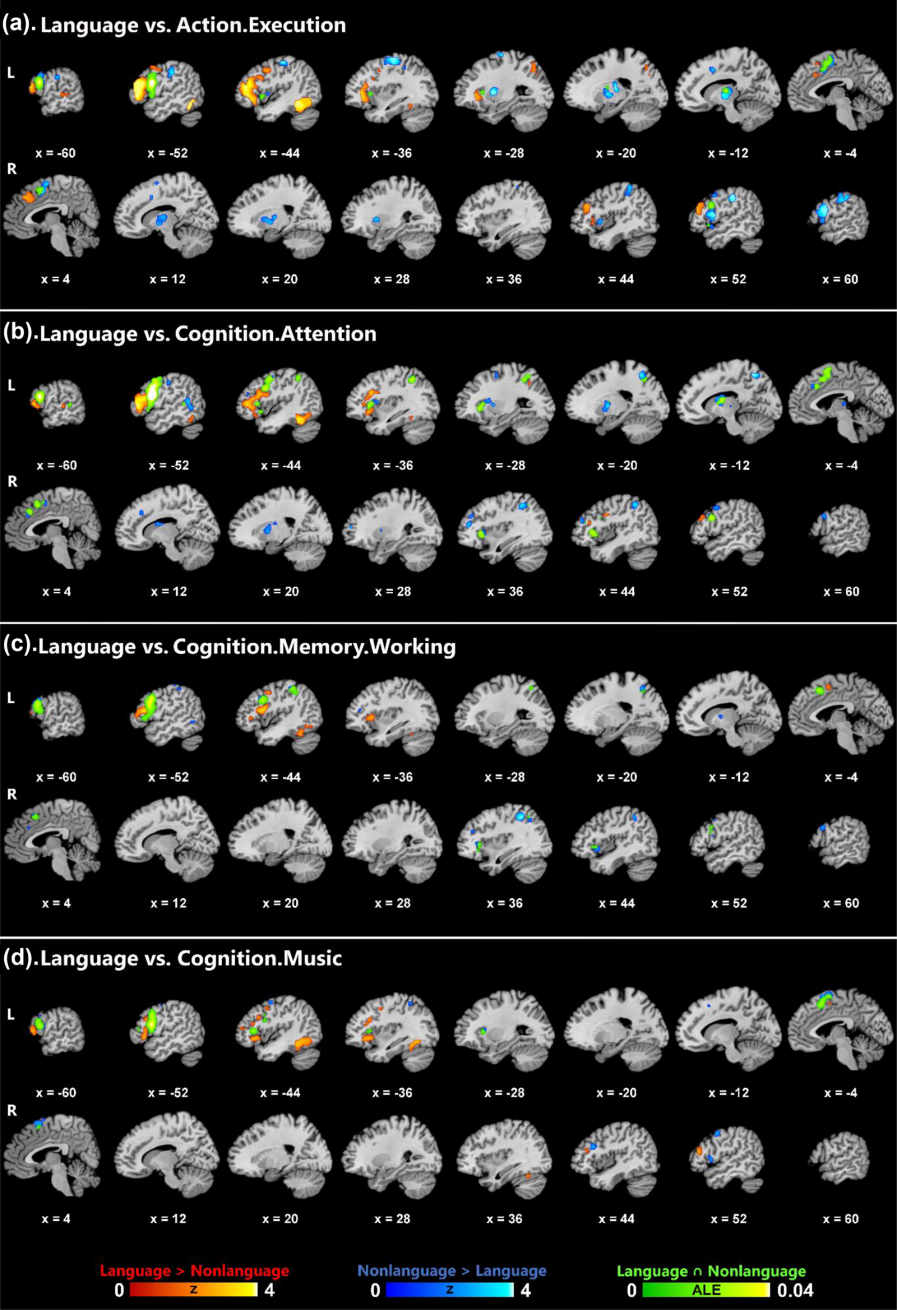
Results of contrast and conjunction analyses between the language domain and four nonlanguage domains for IFGop. Color bars indicate *Z* scores for contrast and ALE scores for conjunction analyses.

The results of contrast and conjunction analyses between language and four nonlanguage domains (action.execution, cognition.attention, cognition.memory.working, and cognition.music) for IFGop are illustrated in Figure 3 (see Supporting Information Tables SI1–4 for a detailed summary of the results), and those between language and cognition.attention domains for IFGtri are illustrated in Supporting Information Figure SI1 and summarized in Supporting Information Table SI5.

Largely in parallel with the results of the main analyses, the domain‐specific (language > nonlanguage) co‐activation network of IFGop compared to each one of the four nonlanguage domains was generally left‐lateralized, spanning left frontal (IFG, MFG, precentral gyrus) and temporal (fusiform gyrus) structures (Figure 3). Likewise, the domain‐specific co‐activation network of IFGtri (language > cognition.attention) closely matched the domain‐specific network identified in the main analyses, involving primarily left frontotemporal regions (IFG, MFG, MTG, fusiform gyrus) (Supporting Information Figure SI1).

The co‐activation network shared between language and each behavioral domain (domain‐general network) for IFGop revealed certain similarities and differences across domains. On the cortical territory, all domain‐general networks involved frontal regions, including IFG and MFG, while they included parietal structures (IPL, SPL, precuneus) only for the cognition.attention and cognition.memory.working domains. Subcortically, particularly for the action.execution and cognition.attention domains, IFGop was associated with nonlanguage co‐activations in the basal ganglia (putamen, globus pallidus, caudate body) and the thalamus (ventral posterior lateral nucleus, ventral lateral nucleus, ventral anterior nucleus). The domain‐general network of IFGop, on the other hand, spanned the left thalamus (ventral anterior/lateral nucleus) only for the conjunction of language with action.execution and cognition.attention, and the left caudate body with cognition.attention.

## 3 DISCUSSION

Using the MACM method, the present study investigated the difference and overlap between the language and nonlanguage networks of the left IFGop, IFGtri, and IFGorb to delineate language‐related domain‐specific (specific to language) and domain‐general (shared between language and nonlanguage domains) co‐activation networks of these ROIs. Thanks to the broad scope of the BrainMap database representing various behavioral domains, it was possible to disentangle the domain‐general and domain‐specific contributions by Broca's area and its functional connectivity network to language processing. The findings revealed a mostly left‐lateralized frontotemporal domain‐specific system for all ROIs. However, the domain‐general network was associated with a frontoparietal system that largely overlaps with the multiple‐demand network. The exploratory analyses showed that the domain‐specific network of IFGop was consistent across four behavioral domains, while its domain‐general network revealed certain differences at cortical and subcortical levels across domains. These findings show that domain‐specificity of Broca's area for language processing arises as part of a functional frontotemporal network, which may recruit additional resources from the domain‐general frontoparietal and subcortical networks depending on task demands.

For all IFG ROIs, the findings revealed a largely left‐lateralized domain‐specific network (obtained using the contrast of language > nonlanguage) that spanned frontal (IFG, MFG) and temporal (fusiform gyrus, STG, MTG) regions, whereas the nonlanguage network (nonlanguage > language) exclusively involved frontoparietal regions. The domain‐general network (language ∩ nonlanguage) identified mostly left‐lateralized, frontoparietal cortices except for a single left temporal lobe cluster, involving STG and MTG, for IFGop. These findings are generally consistent with the accounts that propose a domain‐specific left frontotemporal network including parts of Broca's area that underlie language processing and that are, at least partially, distinct from domain‐general networks (Campbell & Tyler, 2018; Fedorenko & Blank, 2020; Fedorenko et al., 2011). Although it is not possible to pinpoint relative contributions of different linguistic components (syntax, phonology, semantics, etc.) to the domain‐specific functional network of the left IFG based on the present findings, it is probably not justifiable to attribute it solely to syntax as has been done in some previous research (Campbell & Tyler, 2018; Grodzinsky & Friederici, 2006; Grodzinsky & Santi, 2008), given that not only the left IFGop, which has more commonly been involved in syntactic processing (Zaccarella et al., 2017), but also IFGtri and IFGorb, which have often been associated with semantic processing (Hagoort & Indefrey, 2014), revealed domain‐specific networks that survived after subtraction of their respective nonlanguage networks. The present findings are incompatible also with the claims that attribute the role of Broca's area in language processing to its involvement in domain‐general processes including cognitive control (January et al., 2009; Novick et al., 2005, 2010), or representation of complex structural and hierarchical relationships across domains including not only language, but also action and music (Fadiga et al., 2009; Fitch & Martins, 2014). Exploratory analyses also revealed a closely similar left frontotemporal domain‐specific network when the language domain was contrasted with action.execution, cognition.attention, cognition.memory.working, and cognition.music for IFGop, and cognition.attention for IFGtri. This finding suggests that the domain‐specific left frontotemporal network identified in the main analyses was not due to overinfluence of a given behavioral domain, but rather was consistent across different domains, at least for the domains that could be analytically investigated for IFGop (four domains) and IFGtri (one domain).

Given that the large number of experiments included in the nonlanguage analysis were related to various nonlanguage and nonspeech behavioral domains (action, cognition, emotion, interoception, and perception), it was possible to take into account extralinguistic factors that may accompany certain language tasks and that were previously associated with Broca's area such as emotional processing (Belyk et al., 2017), mathematical processing (Maruyama et al., 2012), action processing (Clos et al., 2013; Papitto et al., 2020), working memory (Clos et al., 2013; Makuuchi et al., 2009), cognitive control (Clos et al., 2013; Novick et al., 2005, 2010), and music (Heard & Lee, 2020; Koelsch, 2006). It should be acknowledged upfront that the current study did not undertake a characterization of the nonlanguage network, which, as mentioned above, spans multiple behavioral domains, each of which may have contributed to the obtained results differently. Therefore, the nonlanguage network was treated as a comprehensive, but heterogenous, baseline against which the language‐related co‐activation profile was compared to delineate the domain‐specific network. Nevertheless, the exploratory analyses provided safeguard to a certain extent by showing consistent domain‐specific co‐activation patterns when compared to individual nonlanguage domains, as mentioned in the preceding paragraph, and enabled better characterization of the domain‐general network for specific domains, as discussed below.

Interestingly, the frontal and parietal regions identified here as part of the nonlanguage and domain‐general networks overlap with the frontoparietal network that has been highlighted as a domain‐general system underlying a range of cognitive functions. Specifically, the frontoparietal network has been conceptualized as a control system incorporating various regions involved in cognitive control and decision making (Vincent et al., 2008) and as a multiple‐demand system underlying various cognitive functions that drive intelligent, goal‐directed behavior (Duncan, 2010; Duncan & Owen, 2000). Indeed, previous research associated parts of Broca's area with the domain‐general frontoparietal multiple‐demand network (Fedorenko & Blank, 2020; Fedorenko et al., 2011). Specifically, superior‐posterior parts of the left IFG were found to be shared between domain‐specific (language) and domain‐general (verbal working memory and cognitive control) functions (Fedorenko et al., 2011). Likewise, the present study found overlap between language and nonlanguage networks in similar parts of the left IFG. The left frontal co‐activations in domain‐general networks of IFG in the main analyses were also identified for all individual domains in the exploratory analyses. However, parietal co‐activations (IPL, SPL, precuneus) were found only for the domain‐general networks of IFGop involving cognition.attention and cognition.memory.working. These findings suggest that the domain‐specific left‐frontotemporal system may recruit additional resources from parts of the domain‐general frontoparietal network depending on task demands (e.g., working memory, cognitive control, etc.).

Relatedly, the domain‐general network may also include co‐activations arising from executive control‐related functions that have been associated with bilingual language processing. In particular, bilingual language control and language switching have been associated with an inhibitory control network spanning frontoparietal and subcortical structures (Calabria et al., 2018; Luk et al., 2012; Rodriguez‐Fornells et al., 2006; Sulpizio et al., 2020). Enhanced recruitment of parts of this network in bilingualism has been ascribed to inhibitory control over and competition among lexemes and lexicons, among others (Abutalebi & Green, 2007; Rodriguez‐Fornells et al., 2006). Moreover, neuroimaging studies directly comparing monolingual and bilingual language processing suggest that when faced with increasing cognitive demands, both groups recruit a common domain‐general system, which may be more activated in bilinguals due to competition within and between languages (Abutalebi et al., 2013; Parker Jones et al., 2012). Therefore, domain‐general frontoparietal and subcortical (left thalamus and basal ganglia) systems may play a role in language processing when additional cognitive resources are needed such as when resolving conflicts due to competition among lexical items or languages as in bilingual language processing.

The domain‐general and nonlanguage networks identified in the current study also included several structures that constitute the resting‐state default mode network. The default mode network spans the bilateral parietal (precuneus, IPL), posterior cingulate, medial prefrontal, and medial and lateral temporal cortices and has been associated with the brain's intrinsic activity (Raichle, 2015; van den Heuvel & Hulshoff Pol, 2010). Previous research showed that the precuneus interacts with both the frontoparietal and the default mode networks, potentially playing a crucial role in organizing task‐ and rest‐related brain activity across these two systems (Utevsky et al., 2014). Consistently, the present study identified the precuneus particularly within the nonlanguage network of IFGop and IFGtri, but also within the domain‐specific and domain‐general networks of IFGop. Furthermore, the exploratory analyses showed that the domain‐general network of IFGop spanned the precuneus along with SPL and IPL only for the conjunction of language with cognition.attention and cognition.memory.working out of the four behavioral domains investigated. These findings imply that the precuneus may serve as an interface not only between the two domain‐general (frontoparietal multiple‐demand and default mode) networks, but also between the language and nonlanguage networks of the left IFG.

Another interface region revealed in the present study is the left orbitofrontal cortex, which demonstrated a lateral‐to‐medial gradient for IFGorb with lateral, lateral‐medial, and medial co‐activation patterns for the domain‐specific, domain‐general, and nonlanguage networks, respectively. Indeed, although the nonlanguage co‐activation network of IFGorb included several limbic structures (right cingulate and parahippocampal gyri), the only overlap between language and nonlanguage connectivity of IFGorb was observed in the left orbitofrontal cortex, with the aforementioned lateral‐to‐medial gradient. This finding is consistent with a previous meta‐analysis that associated lateral IFGorb with both emotion and semantics, and medial or opercular IFGorb with emotion alone (Belyk et al., 2017). Taken together, these findings highlight the similarities and differences between the domain‐specific and domain‐general contributors to language processing, which may better be conceptualized as a gradient than a dichotomy.

Although the functional connectivity networks of the ROIs were predominantly cortical, several subcortical structures were also identified. All ROIs showed some subcortical co‐activation primarily within the putamen and/or thalamus as part of their nonlanguage network, while the nonlanguage networks of IFGop also included the bilateral caudate nuclei. However, only IFGop and IFGtri had domain‐specific co‐activation extending to subcortical structures (left thalamus), and only IFGop had domain‐general co‐activation with the left thalamus and putamen. Exploratory analyses showed that the domain‐general network of IFGop spanned the left thalamus (ventral anterior/lateral nucleus) only for conjunction of language with action.execution and cognition.attention, but not with cognition.memory.working or cognition.music. Moreover, the domain‐specific networks of all ROIs showed some co‐activation with the left cerebellum, but this cerebellar involvement was part of a larger cluster overlapping with the fusiform gyrus, whereas only IFGop exhibited distinct co‐activation with the right cerebellum within its domain‐specific network. The exploratory analyses also identified this domain‐specific co‐activation of the right cerebellum with IFGop when contrasted with action.execution, cognition.attention, and cognition.music. Previous research associated the cerebello‐basal ganglia‐thalamo‐cortical system with a broad range of cognitive and sensorimotor functions including language (Bostan & Strick, 2010, 2018; Bostan et al., 2013; Caligiore et al., 2017; Ford et al., 2013; Tomasi & Volkow, 2012). Accordingly, the present findings also underline the domain‐specific and domain‐general aspects of this loop for language processing.

Several potential limitations should be addressed. First, the experiments included in the analyses of the language network of IFG subdivisions pertain to multiple language components (syntax, semantics, phonology, orthography, speech), tasks (e.g., comprehension, production), and modalities (e.g., visual, auditory). This was intended as a generalization from specific tasks to language processing in general. Nevertheless, divergence, but also convergence, in the neural representation of different language functions were shown in previous research, for example, between syntax and semantics (Hagoort & Indefrey, 2014; Rodd et al., 2015). Therefore, the domain‐specific network identified in this study likely consists of subnetworks that may at least partially be dissociable from each other. Relatedly, it could be argued that certain language subdomains, such as syntax, are more domain‐specific than others (Campbell & Tyler, 2018; Grodzinsky & Friederici, 2006; Grodzinsky & Santi, 2008). Given that the domain‐specific versus domain‐general issue was addressed here from the viewpoint of subdivisions of Broca's area and their co‐activation networks, and that the number of experiments identified for each language subdomain (syntax, semantics, phonology, orthography, speech) for these ROIs did not allow computing MACM separately for the majority of those subdomains, only the overall language networks were explored. Future MACM studies may tease apart language‐specific co‐activation patterns of subdivisions of Broca's area across language subdomains by extending study inclusion beyond BrainMap and with the availability of more neuroimaging research.

Another potential limitation concerns ROI definition. The functional and anatomical heterogeneity of Broca's area clearly extends beyond the anatomical labels of IFGop, IFGtri, and IFGorb. For example, previous research identified functional division of labor within IFGop along the anterior–posterior axis (Clos et al., 2013; Papitto et al., 2020), and within IFGorb along the lateral–medial axis (Belyk et al., 2017). However, subdivision of Broca's area based on Brodmann Areas is still applicable to many neurocognitive accounts of language (Friederici, 2002, 2011, 2012; Hagoort, 2005b, 2013, 2016; Hickok & Poeppel, 2004, 2007; Poeppel et al., 2012; Ullman, 2001, 2004, 2016). Also, the probabilistic atlas used here in ROI definition (Amunts et al., 2020) is based on the brain's cytoarchitecture, which is tightly linked to brain functions and connectivity patterns (Amunts et al., 2020; Goulas et al., 2018; Wojtasik et al., 2020). Moreover, given that the atlas is probabilistic, it allows for characterizing the relevant regions consistently (Robinson et al., 2010), and attenuates interindividual variations in anatomy (Amunts et al., 2020; Fedorenko & Blank, 2020). Indeed, to ensure that ROIs represented the intended brain regions, they were thresholded with minimum probabilities greater than .48. Finally, although three subdivisions of IFG are scrutinized with regard to their domain‐specific functional co‐activation network, no claim has been made concerning language‐specificity of any given IFG subregion. This is because the present study does not attempt a functional fractionation of Broca's area, and merely examines the language‐specific functional *network* of Broca's area, a region which has been conceptualized anatomically to include different combinations of IFG subdivisions. Indeed, in parallel with the literature, all three IFG subdivisions examined here exhibited domain‐specific, language‐related functional networks, albeit with certain differences. Therefore, this exploration does not address whether there are any language‐specific subregions within Broca's area in general or within its subdivisions. Future investigations of language‐specific subregions within Broca's area and in other brain regions may benefit from more detailed parcellation techniques in ROI definition including connectivity‐based parcellation (Fan et al., 2016; Wang et al., 2015) and multiple receptor mapping (Amunts et al., 2010).

## 4 CONCLUSIONS

The present findings show that the language‐related domain‐specific functional network of Broca's area spans mainly left‐lateralized frontotemporal regions including the middle and inferior frontal cortices as well as anterior, posterior, and inferior temporal cortices. Broca's area was also associated with domain‐general frontoparietal and subcortical (thalamus and basal ganglia) networks shared between language and nonlanguage domains. The findings suggest that Broca's area, or Broca's complex in a broad sense, spanning the opercular, triangular and orbital parts of the left IFG, exhibits language‐specificity as part of a functional connectivity network involving a left frontotemporal system, which recruits domain‐general resources from frontoparietal multiple‐demand and subcortical (thalamus and basal ganglia) networks based on task demands. Application of MACM to compare divergence and convergence between domain‐specific and domain‐general functional networks of brain regions, as in the present study, offers significant potential for explorations of specific and shared networks for other language‐related regions and other behavioral domains.

### PEER REVIEW

The peer review history for this article is available at https://publons.com/publon/10.1002/brb3.3046.

## Supporting information

Table SI1. Contrast and conjunction results of the language and action.execution domains for IFGop.
Table SI2. Contrast and conjunction results of the language and cognition.attention domains for IFGop.
Table SI3. Contrast and conjunction results of the language and cognition.memory.working domains for IFGop.
Table SI4. Contrast and conjunction results of the language and cognition.music domains for IFGop.
Table SI5. Contrast and conjunction results of the language and cognition.attention domains for IFGtri.
Figure SI1. Contrast and conjunction results of the language and attention (nonlanguage) domains for IFGtri.

## Data Availability

All data and analysis files associated with the study are available at https://doi.org/10.17632/tfg4pryhf9.1.

## NOTES

1 The left‐hemispheric ROIs and the neuroimaging experiments included in the language analyses in the present research were the same as a previous study on language‐related connectivity of bilateral IFG (Bulut, 2022a). However, differently from that study, the present study included a nonlanguage contrast and compared functional networks of the left IFG subdivisions for language and nonlanguage domains in contrast and conjunction analyses in an attempt to identify language‐related domain‐specific and domain‐general circuitry of these ROIs.

2 Minimum and maximum probabilities are given in the color bars in Figure 1. The mean probabilities (SD) and sizes of the ROIs were as follows: IFGop: 0.77 (0.09), 2409 mm^3^; IFGtri: 0.64 (0.12), 2363 mm^3^; IFGorb: 0.68 (0.12), 2353 mm^3^.

3 In the nonlanguage search with IFGop as the ROI, one duplicate experiment (Rypma et al., 2001), which reported the same coordinates of activation as another experiment (Rypma et al., 2001), was identified and eliminated from both Table 1 and the meta‐analyses.
