# Supplementary material for: Domain‐general and domain‐specific functional networks of Broca's area underlying language processing
Source: Brain Behav. 2023 May 3;13(7):e3046. doi: 10.1002/brb3.3046 (PMC10338813; doi:10.1002/brb3.3046)
Supplement: Supplementary file 1 — Table SI1. Contrast and conjunction results of the language and action.execution domains for IFGop. Table SI2. Contrast and conjunction results of the language and cognition.attention domains for IFGop. Table SI3. Contrast and conjunction results of the language and cognition.memory.working domains for IFGop. Table SI4. Contrast and conjunction results of the language and cognition.music domains for IFGop. Table SI5. Contrast and conjunction results of the language and cognition.attention domains for IFGtri. Figure SI1. Contrast and conjunction results of the language and attention (nonlanguage) domains for IFGtri. [file BRB3-13-e3046-s001.docx]

**Supporting Information**

**for**

**Domain-general and domain-specific functional networks of Broca’s area underlying language processing**

This Supporting Information provides details on the exploratory analyses. The results of contrast and conjunction analyses between language and four nonlanguage domains (action.execution, cognition.attention, cognition.memory.working, and cognition.music) for IFGop are summarized in Tables SI1-4 below. The results of contrast and conjunction analyses between language and cognition.attention domains for IFGtri are illustrated in Figure SI1 and summarized in Table SI5 below. Please refer to *ALE analyses* under *Materials and methods* for further details on the analyses. The data associated with the following results are available at <https://doi.org/10.17632/tfg4pryhf9.1>.

Table SI1. Contrast and conjunction results of the language and action.execution domains for IFGop.

| **Cluster** | **Anatomical Label**  (Nearest Gray Matter within 5mm) | **BA** | **MNI Coordinates** | | | **Z / ALE** | **Cluster Size** (mm^3^) |
| --- | --- | --- | --- | --- | --- | --- | --- |
|  |  |  | x | y | z |  |  |
| IFGop Language > Action.Execution | | | | | | Z |  |
| 1 | L IFG | 46 | -50.5 | 32.6 | 11.4 | 3.89 | 16448 |
|  | L MFG | 46 | -40.3 | 32.4 | 10.4 | 2.85 |  |
|  | L IFG | 9 | -46 | 14 | 28 | 2.54 |  |
| 2 | L Fusiform Gyrus | 37 | -47.1 | -52.4 | -16 | 3.89 | 5616 |
|  | L ITG | 19 | -47.3 | -60.7 | -6 | 3.72 |  |
|  | L Fusiform Gyrus | 37 | -45.3 | -68 | -11.2 | 3.35 |  |
| 3 | R MFG | 9 | 48 | 35 | 26 | 3.12 | 2360 |
|  | R MFG | 46 | 48 | 34 | 20 | 2.99 |  |
| 4 | R FGmed | 8 | 8 | 32 | 44 | 2.86 | 2008 |
|  | R Cingulate Gyrus | 32 | 8 | 26 | 38 | 2.76 |  |
|  | L Cingulate Gyrus | 32 | 0 | 28 | 34 | 2.52 |  |
|  | L Cingulate Gyrus | 32 | -4 | 26 | 34 | 2.15 |  |
|  | L SFG | 8 | 0 | 28 | 52 | 1.92 |  |
| 5 | L Cuneus | 7 | -22 | -76 | 38 | 2.93 | 1496 |
|  | L Precuneus | 19 | -26 | -76 | 38 | 2.83 |  |
|  | L Precuneus | 19 | -26 | -74 | 42 | 2.78 |  |
|  | L Precuneus | 19 | -26 | -70 | 36 | 2.66 |  |
|  | L Precuneus | 7 | -26 | -72 | 46 | 2.28 |  |
|  | L Precuneus | 19 | -30 | -68 | 52 | 2.08 |  |
| 6 | L MFG | 6 | -44 | 4 | 42 | 2.70 | 1464 |
| 7 | L MTG | 22 | -56 | -38 | 0 | 2.23 | 584 |
|  | L MTG | 21 | -60 | -30 | -2 | 2.05 |  |
| 8 | R IFG | 47 | 46 | 22 | -4 | 1.98 | 224 |
|  | R Insula | 13 | 46 | 20 | 0 | 1.79 |  |
|  | R Insula | 13 | 42 | 24 | 8 | 1.79 |  |
| 9 | L SFG | 6 | -2 | 24 | 56 | 1.90 | 120 |
|  | L SFG | 6 | -4 | 18 | 52 | 1.89 |  |
| 10 | R Culmen (Cerebellum) |  | 42 | -60 | -26 | 1.92 | 112 |
| IFGop Action.Execution > Language | | | | | | Z |  |
| 1 | R IFG | 44 | 58.1 | 8.5 | 15.6 | 3.89 | 5888 |
|  | R Precentral Gyrus | 44 | 62 | 10 | 4 | 3.16 |  |
|  | R Precentral Gyrus | 6 | 56 | 8 | 32 | 2.57 |  |
|  | R STG | 22 | 54 | 10 | -8 | 2.27 |  |
| 2 | L Putamen |  | -25.5 | -4.5 | 5 | 3.89 | 5176 |
|  | L Thalamus |  | -10.8 | -13.5 | -3.4 | 3.72 |  |
|  | L Ventral Posterior Lateral Nucleus (Thalamus) |  | -16 | -16 | 6 | 3.54 |  |
| 3 | L Precentral Gyrus | 4 | -36.9 | -21.1 | 58.4 | 3.89 | 3856 |
| 4 | R Lateral Globus Pallidus |  | 21.6 | -0.8 | 0 | 3.04 | 3272 |
|  | R Putamen |  | 26 | 4 | 6 | 3.43 |  |
|  | R Putamen |  | 26 | 12 | 6 | 3.29 |  |
|  | R Lateral Globus Pallidus |  | 22 | -4 | -2 | 3.04 |  |
| 5 | R Cingulate Gyrus | 24 | 4 | 3 | 48 | 3.89 | 2984 |
|  | R FGmed | 6 | 3.6 | -5.6 | 62 | 3.43 |  |
|  | L Paracentral Lobule | 31 | -6 | -6 | 50 | 2.82 |  |
|  | R Cingulate Gyrus | 24 | 10 | 6 | 40 | 2.69 |  |
|  | R Cingulate Gyrus | 24 | 8 | 8 | 44 | 2.63 |  |
| 6 | R Postcentral Gyrus | 2 | 53.6 | -25.4 | 38.6 | 3.89 | 2088 |
|  | R Postcentral Gyrus | 2 | 62 | -22 | 38 | 3.54 |  |
|  | R Postcentral Gyrus | 3 | 62 | -18 | 41 | 3.06 |  |
| 7 | L IFG | 9 | -60 | 7 | 21 | 3.54 | 2072 |
|  | L Precentral Gyrus | 6 | -58 | 8 | 30 | 2.83 |  |
|  | L Precentral Gyrus | 44 | -52 | 5 | 9 | 1.82 |  |
| 8 | L Postcentral Gyrus | 2 | -50.5 | -25.3 | 37.3 | 3.72 | 1448 |
|  | L IPL | 40 | -59.3 | -24 | 28.7 | 2.79 |  |
| 9 | R IPL | 40 | 42 | -42 | 48 | 3.54 | 1024 |
|  | R IPL | 40 | 44 | -40 | 56 | 3.16 |  |
|  | R IPL | 40 | 40 | -40 | 54 | 3.04 |  |
|  | R IPL | 40 | 40 | -36 | 58 | 2.99 |  |
|  | R IPL | 40 | 38 | -40 | 58 | 2.86 |  |
| 10 | L Cingulate Gyrus | 24 | -8.7 | 8 | 43.3 | 3.43 | 792 |
|  | L Cingulate Gyrus | 24 | -10 | 4 | 44 | 3.35 |  |
| 11 | R Precentral Gyrus | 6 | 50 | 6 | 49 | 2.81 | 256 |
| 12 | L Insula | 13 | -40 | 12 | -2 | 2.15 | 192 |
| 13 | L Insula | 13 | -44 | 0 | 2 | 2.42 | 144 |
| 14 | L Supramarginal Gyrus | 40 | -36 | -38 | 44 | 2.34 | 136 |
| IFGop Language ∩ Action.Execution | | | | | | ALE |  |
| 1 | L IFG | 44 | -54 | 8 | 20 | 0.064 | 5752 |
|  | L STG | 22 | -52 | 6 | 2 | 0.019 |  |
| 2 | L FGmed | 6 | 0 | 4 | 54 | 0.033 | 2352 |
|  | L FGmed | 6 | 0 | 0 | 60 | 0.024 |  |
|  | L Cingulate Gyrus | 24 | -6 | 10 | 44 | 0.022 |  |
| 3 | L Claustrum |  | -32 | 18 | 4 | 0.023 | 928 |
|  | L Insula | 13 | -42 | 12 | -2 | 0.02 |  |
| 4 | R IFG | 9 | 54 | 10 | 26 | 0.029 | 688 |
| 5 | L Ventral Lateral Nucleus (Thalamus) |  | -12 | -14 | 6 | 0.026 | 392 |
| 6 | R IFG | 47 | 56 | 16 | -6 | 0.02 | 128 |

Note: MNI Coordinates correspond to cluster peaks, and anatomical labels indicate gray matter nearest to the cluster peaks. Please refer to the online data repository for cluster analyses with full reports of structures included in each cluster. L: Left, R: Right, FGmed: Medial frontal gyrus, IFG: Inferior frontal gyrus, IPL: Inferior parietal lobule, ITG: Inferior temporal gyrus, MFG: Middle frontal gyrus, MTG: Middle temporal gyrus, SFG: Superior frontal gyrus, SPL: Superior parietal lobule, STG: Superior temporal gyrus.

Table SI2. Contrast and conjunction results of the language and cognition.attention domains for IFGop.

| **Cluster** | **Anatomical Label**  (Nearest Gray Matter within 5mm) | **BA** | **MNI Coordinates** | | | **Z / ALE** | **Cluster Size** (mm^3^) |
| --- | --- | --- | --- | --- | --- | --- | --- |
|  |  |  | x | y | z |  |  |
| IFGop Language > Cognition.Attention | | | | | | Z |  |
| 1 | L IFG | 46 | -51 | 34 | 7.4 | 3.43 | 11720 |
|  | L IFG | 46 | -52.7 | 32.3 | 11.5 | 3.72 |  |
|  | L MFG | 9 | -40 | 16 | 22 | 3.19 |  |
|  | L MFG | 9 | -39 | 18 | 26 | 3.16 |  |
|  | L Insula | 13 | -42 | 6 | 20 | 2.95 |  |
|  | L Extra-Nuclear | 47 | -36 | 22 | -8 | 2.72 |  |
|  | L STG | 22 | -60 | 12 | 0 | 2.41 |  |
| 2 | L Culmen (Cerebellum) |  | -45 | -51 | -21 | 3.89 | 3096 |
|  | L Culmen (Cerebellum) |  | -45 | -47 | -24 | 3.72 |  |
|  | L Fusiform Gyrus | 37 | -44 | -44 | -20 | 3.54 |  |
| 3 | R MFG | 46 | 44 | 28 | 16 | 2.28 | 696 |
|  | R MFG | 9 | 56 | 26 | 28 | 2.18 |  |
|  | R MFG | 46 | 46 | 24 | 14 | 2.11 |  |
| 4 | L STG |  | -60 | -28 | 2 | 2.09 | 184 |
| 5 | R Culmen (Cerebellum) |  | 42 | -58 | -26 | 2.05 | 176 |
|  | R Tuber (Cerebellum) |  | 42 | -64 | -26 | 1.86 |  |
| 6 | R Precentral Gyrus | 6 | 44 | 0 | 30 | 2.23 | 144 |
| 7 | L Angular Gyrus | 39 | -30 | -60 | 42 | 2.01 | 136 |
| IFGop Cognition.Attention > Language | | | | | | Z |  |
| 1 | L Lateral Globus Pallidus |  | -20.7 | -2.7 | 2 | 3.89 | 2800 |
|  | L Putamen |  | -26 | -2 | 2 | 3.54 |  |
|  | L Ventral Anterior Nucleus (Thalamus) |  | -14 | -2 | 8 | 3.19 |  |
|  | L Caudate Body |  | -10 | 2 | 16 | 3.01 |  |
|  | L Ventral Lateral Nucleus (Thalamus) |  | -16 | -12 | 16 | 1.93 |  |
| 2 | R Putamen |  | 26 | 4 | 6 | 3.09 | 1896 |
|  | R Caudate Body |  | 14 | -2 | 16 | 2.99 |  |
|  | R Ventral Lateral Nucleus (Thalamus) |  | 16 | -12 | 16 | 2.28 |  |
|  | R Caudate Body |  | 16 | 12 | 4 | 2.21 |  |
| 3 | L Precuneus | 7 | -14.7 | -59 | 54.3 | 3.89 | 1608 |
| 4 | R IPL | 40 | 39.9 | -46.7 | 47.9 | 3.89 | 1424 |
| 5 | R SFG | 10 | 30 | 50 | 12 | 3.43 | 1320 |
|  | R MFG | 10 | 32 | 46 | 14 | 3.35 |  |
|  | R SFG | 9 | 44 | 42 | 22 | 2.62 |  |
|  | R MFG | 9 | 40 | 40 | 30 | 2.49 |  |
| 6 | R Cingulate Gyrus | 32 | 10 | 30 | 36 | 2.13 | 896 |
|  | L FGmed | 8 | -4 | 32 | 38 | 2.07 |  |
|  | R Cingulate Gyrus | 32 | 4 | 30 | 28 | 2.04 |  |
| 7 | L STG | 39 | -52 | -52 | 8 | 3.35 | 840 |
| 8 | L MFG | 6 | -52 | 8 | 40 | 2.95 | 824 |
| 9 | R IFG | 9 | 58 | 10 | 32 | 2.40 | 704 |
| 10 | R Claustrum |  | 40 | 14 | -2 | 2.65 | 656 |
| 11 | L Putamen |  | -28 | 6 | 10 | 2.42 | 592 |
|  | L Claustrum |  | -28 | 14 | 12 | 2.38 |  |
|  | L Insula | 13 | -36 | 8 | 8 | 2.13 |  |
| 12 | R Precentral Gyrus | 6 | 52 | 6 | 44 | 2.34 | 336 |
| 13 | L Precentral Gyrus | 6 | -48 | -2 | 54 | 2.25 | 312 |
| 14 | L Thalamus |  | -8 | -22 | 2 | 2.40 | 264 |
| 15 | L IPL | 40 | -34 | -50 | 46 | 2.16 | 256 |
| 16 | L Postcentral Gyrus | 3 | -52 | -18 | 42 | 2.42 | 248 |
| 17 | L MFG | 6 | -26 | -4 | 54 | 2.55 | 232 |
| 18 | L MFG | 46 | -42 | 38 | 22 | 2.11 | 176 |
| 19 | L FGmed | 6 | 2 | -2 | 50 | 2.45 | 160 |
| IFGop Language ∩ Cognition.Attention | | | | | |  |  |
| 1 | L IFG | 44 | -54 | 10 | 20 | 0.095 | 10600 |
|  | L IFG | 9 | -50 | 10 | 32 | 0.054 |  |
|  | L Precentral Gyrus | 6 | -50 | 4 | 44 | 0.041 |  |
|  | L IFG | 47 | -46 | 16 | -8 | 0.025 |  |
| 2 | L FGmed | 32 | 0 | 14 | 46 | 0.030 | 2824 |
|  | L FGmed | 6 | 0 | 2 | 52 | 0.029 |  |
|  | L FGmed | 6 | -4 | 8 | 62 | 0.025 |  |
| 3 | L IPL | 40 | -32 | -52 | 48 | 0.033 | 2624 |
|  | L IPL | 40 | -34 | -48 | 44 | 0.030 |  |
|  | L Precuneus | 7 | -22 | -62 | 46 | 0.028 |  |
|  | L IPL | 40 | -40 | -48 | 50 | 0.028 |  |
| 4 | R Insula | 13 | 40 | 22 | 0 | 0.036 | 2096 |
| 5 | L Insula | 13 | -32 | 22 | 2 | 0.033 | 2040 |
|  | L Insula | 13 | -32 | 20 | 6 | 0.033 |  |
|  | L IFG | 45 | -42 | 22 | 6 | 0.024 |  |
| 6 | R Cingulate Gyrus | 32 | 6 | 26 | 34 | 0.031 | 1048 |
|  | L Cingulate Gyrus | 32 | -4 | 24 | 36 | 0.026 |  |
|  | R FGmed | 8 | 6 | 32 | 38 | 0.022 |  |
| 7 | L Ventral Anterior Nucleus (Thalamus) |  | -12 | -6 | 12 | 0.032 | 872 |
|  | L Caudate Body |  | -12 | 2 | 8 | 0.022 |  |
| 8 | R IFG | 9 | 52 | 10 | 26 | 0.033 | 872 |
| 9 | L MTG | 21 | -54 | -46 | 6 | 0.023 | 408 |

Note: Please see Note under Table SI1 for definition of abbreviations.

Table SI3. Contrast and conjunction results of the language and cognition.memory.working domains for IFGop.

| **Cluster** | **Anatomical Label**  (Nearest Gray Matter within 5mm) | **BA** | **MNI Coordinates** | | | **Z / ALE** | **Cluster Size** (mm^3^) |
| --- | --- | --- | --- | --- | --- | --- | --- |
|  |  |  | x | y | z |  |  |
| IFGop Language > Cognition.Memory.Working | | | | | | Z |  |
| 1 | L IFG | 45 | -52 | 24 | 14 | 3.89 | 5528 |
|  | L IFG | 45 | -48 | 22 | 14 | 3.72 |  |
|  | L Insula | 13 | -44 | 14 | 14 | 3.43 |  |
|  | L IFG | 9 | -44 | 8 | 18 | 3.19 |  |
|  | L Claustrum |  | -34 | 18 | 2 | 2.53 |  |
|  | L IFG | 45 | -40 | 28 | -2 | 2.20 |  |
| 2 | L Culmen (Cerebellum) |  | -44 | -52 | -20 | 2.35 | 920 |
|  | L Culmen (Cerebellum) |  | -44 | -48 | -26 | 2.28 |  |
| 3 | L Precentral Gyrus | 6 | -44 | 2 | 44 | 2.11 | 520 |
| 4 | L FGmed | 6 | -6 | 2 | 52 | 2.10 | 432 |
| 5 | L Fusiform Gyrus | 37 | -44 | -62 | -12 | 1.85 | 152 |
|  | L Fusiform Gyrus | 19 | -42 | -70 | -12 | 1.74 |  |
| IFGop Cognition.Memory.Working > Language | | | | | | Z |  |
| 1 | L Precentral Gyrus | 6 | -58 | 10 | 34 | 3.29 | 1976 |
|  | L MFG | 9 | -47 | 12 | 38 | 2.58 |  |
| 2 | R IPL | 40 | 37.9 | -45.2 | 47 | 3.89 | 1520 |
| 3 | R IFG | 9 | 60 | 10 | 32 | 2.60 | 648 |
|  | R IFG | 44 | 56 | 12 | 20 | 2.11 |  |
| 4 | L IPL | 40 | -46 | -32 | 54 | 2.97 | 552 |
| 5 | L Precuneus | 7 | -18 | -60 | 52 | 2.83 | 448 |
| 6 | L SFG | 6 | 2 | 20 | 50 | 2.40 | 288 |
| 7 | R Insula | 13 | 40 | 14 | -6 | 2.68 | 280 |
| 8 | R Insula | 13 | 34 | 30 | 2 | 2.23 | 224 |
| 9 | L Lateral Globus Pallidus |  | -16 | -4 | 4 | 2.18 | 144 |
| 10 | L MFG | 46 | -38 | 38 | 16 | 2.28 | 136 |
| 11 | R Cingulate Gyrus | 32 | 4 | 28 | 28 | 2.09 | 136 |
| 12 | L ITG | 37 | -54 | -58 | -4 | 2.40 | 128 |
| 13 | R MFG | 9 | 36 | 38 | 24 | 2.05 | 120 |
| 14 | R MFG | 6 | 48 | 6 | 42 | 2.07 | 120 |
| 15 | R Angular Gyrus | 39 | 38 | -58 | 44 | 1.95 | 104 |
| IFGop Language ∩ Cognition.Memory.Working | | | | | | ALE |  |
| 1 | L IFG | 9 | -56 | 14 | 26 | 0.041 | 7992 |
|  | L MFG | 9 | -50 | 10 | 34 | 0.040 |  |
| 2 | L SFG | 6 | -2 | 18 | 48 | 0.030 | 1576 |
| 3 | L IPL | 40 | -46 | -38 | 50 | 0.023 | 1208 |
|  | L IPL | 40 | -42 | -44 | 44 | 0.016 |  |
| 4 | R Insula | 13 | 46 | 16 | -6 | 0.019 | 1032 |
|  | R Insula | 13 | 36 | 26 | 2 | 0.019 |  |
|  | R Insula | 13 | 42 | 22 | -4 | 0.016 |  |
|  | R Insula | 13 | 34 | 28 | -8 | 0.015 |  |
| 5 | L SPL | 7 | -24 | -62 | 52 | 0.022 | 584 |
| 6 | R IFG | 9 | 56 | 14 | 28 | 0.019 | 248 |
|  | R IFG | 9 | 54 | 14 | 22 | 0.015 |  |
| 7 | R Precuneus | 19 | 34 | -58 | 44 | 0.017 | 216 |

Note: Please see Note under Table SI1 for definition of abbreviations.

Table SI4. Contrast and conjunction results of the language and cognition.music domains for IFGop.

| **Cluster** | **Anatomical Label**  (Nearest Gray Matter within 5mm) | **BA** | **MNI Coordinates** | | | **Z / ALE** | **Cluster Size** (mm^3^) |
| --- | --- | --- | --- | --- | --- | --- | --- |
|  |  |  | x | y | z |  |  |
| IFGop Language > Cognition.Music | | | | | | Z |  |
| 1 | L Fusiform Gyrus | 37 | -39.3 | -43.3 | -14 | 3.43 | 3984 |
|  | L Fusiform Gyrus | 37 | -38 | -60 | -8 | 3.29 |  |
| 2 | L IFG | 47 | -50 | 22 | -8 | 3.19 | 3976 |
|  | L IFG | 47 | -39 | 30 | -8 | 3.06 |  |
|  | L IFG | 47 | -40 | 26 | -6 | 3.04 |  |
|  | L IFG | 45 | -60 | 22 | 4 | 2.69 |  |
| 3 | No Gray Matter found |  | -36 | 26 | 20 | 2.34 | 1528 |
|  | L MFG | 9 | -36 | 20 | 28 | 2.29 |  |
|  | L IFG | 9 | -52 | 18 | 26 | 2.25 |  |
|  | L MFG | 46 | -46 | 28 | 18 | 1.95 |  |
| 4 | R MFG | 46 | 52 | 32 | 14 | 2.57 | 864 |
| 5 | L MFG | 6 | -40 | 6 | 44 | 2.28 | 408 |
| 6 | R Culmen (Cerebellum) |  | 37 | -56 | -24 | 2.14 | 320 |
|  | R Culmen (Cerebellum) |  | 38 | -60 | -22 | 2.06 |  |
| 7 | L MFG | 10 | -44 | 48 | 12 | 1.87 | 112 |
|  | L MFG | 46 | -40 | 40 | 10 | 1.78 |  |
| 8 | L FGmed | 6 | 0 | 2 | 50 | 1.85 | 104 |
|  | L FGmed | 6 | -5 | 1 | 53 | 1.75 |  |
| IFGop Cognition.Music > Language | | | | | | Z |  |
| 1 | L FGmed | 6 | -2 | 10 | 66 | 3.35 | 2584 |
|  | L FGmed | 6 | 0 | 4 | 66 | 3.09 |  |
|  | R SFG | 6 | 6 | 18 | 62 | 2.81 |  |
|  | L FGmed | 6 | -10 | 14 | 50 | 2.13 |  |
| 2 | L IFG | 44 | -56 | 8 | 12 | 2.79 | 824 |
| 3 | R Insula | 13 | 51 | 10 | 3 | 2.67 | 432 |
| 4 | L Precentral Gyrus | 6 | -46 | -2 | 54 | 2.43 | 400 |
| 5 | R MFG | 9 | 42 | 22 | 24 | 2.83 | 312 |
| 6 | L MFG | 6 | -56 | 10 | 38 | 2.17 | 312 |
| 7 | L Claustrum |  | -26 | 16 | 8 | 2.18 | 256 |
| 8 | R Precentral Gyrus | 6 | 52 | 4 | 46 | 2.99 | 224 |
| 9 | L IPL | 40 | -36 | -48 | 52 | 1.88 | 112 |
| IFGop Language ∩ Cognition.Music | | | | | | ALE |  |
| 1 | L IFG | 44 | -54 | 10 | 16 | 0.049 | 6016 |
|  | L IFG | 9 | -50 | 8 | 32 | 0.033 |  |
| 2 | L SFG | 6 | -4 | 2 | 66 | 0.024 | 2328 |
|  | L SFG | 6 | -6 | 14 | 50 | 0.023 |  |
|  | L SFG | 6 | -4 | 14 | 54 | 0.023 |  |
|  | L SFG | 6 | 0 | 12 | 60 | 0.022 |  |
| 3 | L IFG | 13 | -42 | 26 | 6 | 0.023 | 1832 |
|  | L Claustrum |  | -30 | 18 | 6 | 0.020 |  |
|  | L MFG | 46 | -54 | 32 | 14 | 0.013 |  |

Note: Please see Note under Table SI1 for definition of abbreviations.

Table SI5. Contrast and conjunction results of the language and cognition.attention domains for IFGtri.

| **Cluster** | **Anatomical Label**  (Nearest Gray Matter within 5mm) | **BA** | **MNI Coordinates** | | | **Z / ALE** | **Cluster Size** (mm^3^) |
| --- | --- | --- | --- | --- | --- | --- | --- |
|  |  |  | x | y | z |  |  |
| IFGtri Language > Cognition.Attention | | | | | | Z |  |
| 1 | L IFG | 47 | -46.7 | 32 | -15.3 | 3.89 | 4224 |
|  | L IFG | 47 | -44 | 30 | -10 | 3.72 |  |
| 2 | L MFG | 46 | -44.7 | 23.1 | 16.9 | 3.89 | 4088 |
|  | L Insula | 13 | -46 | 4 | 14 | 3.01 |  |
| 3 | L Culmen (Cerebellum) |  | -38 | -58 | -20 | 3.09 | 3248 |
|  | L Fusiform Gyrus | 37 | -50 | -52 | -18 | 2.75 |  |
|  | L Fusiform Gyrus | 19 | -46 | -72 | -8 | 2.62 |  |
|  | L Declive (Cerebellum) |  | -40 | -68 | -14 | 2.50 |  |
|  | L Fusiform Gyrus | 19 | -40 | -70 | -10 | 2.40 |  |
| 4 | L MTG | 21 | -50 | -36 | -4 | 3.72 | 2248 |
|  | L MTG | 21 | -57.6 | -32.4 | -4.8 | 3.43 |  |
|  | L MTG | 21 | -60 | -38 | -4 | 3.29 |  |
|  | L MTG | 21 | -58 | -28 | -8 | 3.19 |  |
| 5 | L MFG | 6 | -46 | 6 | 48 | 3.09 | 1640 |
| 6 | L MTG | 22 | -54 | -14 | -11 | 3.29 | 992 |
|  | L MTG | 21 | -56 | -8 | -18 | 2.14 |  |
| 7 | L FGmed | 6 | -12 | 8 | 60 | 2.88 | 920 |
|  | L Cingulate Gyrus | 24 | -6 | 8 | 48 | 1.98 |  |
| 8 | L IFG | 45 | -58 | 18 | 0 | 2.54 | 504 |
| 9 | R Insula | 13 | 34 | 28 | -6 | 1.85 | 104 |
| IFGtri Cognition.Attention > Language | | | | | | Z |  |
| 1 | L IPL | 40 | -33 | -48 | 43 | 2.09 | 208 |
|  | L IPL | 7 | -32 | -54 | 48 | 1.81 |  |
| 2 | L MTG | 21 | -64 | -54 | 4 | 2.24 | 152 |
| 3 | L MFG | 9 | -52 | 36 | 26 | 2.04 | 152 |
|  | L MFG | 9 | -46 | 38 | 24 | 2.04 |  |
| IFGtri Language ∩ Cognition.Attention | | | | | | ALE |  |
| 1 | L IFG | 46 | -52 | 30 | 8 | 0.063 | 8656 |
|  | L MFG | 46 | -50 | 28 | 24 | 0.040 |  |
|  | L IFG | 6 | -46 | 6 | 32 | 0.024 |  |
| 2 | L SFG | 6 | -4 | 18 | 52 | 0.023 | 824 |

Note: Please see Note under Table SI1 for definition of abbreviations.


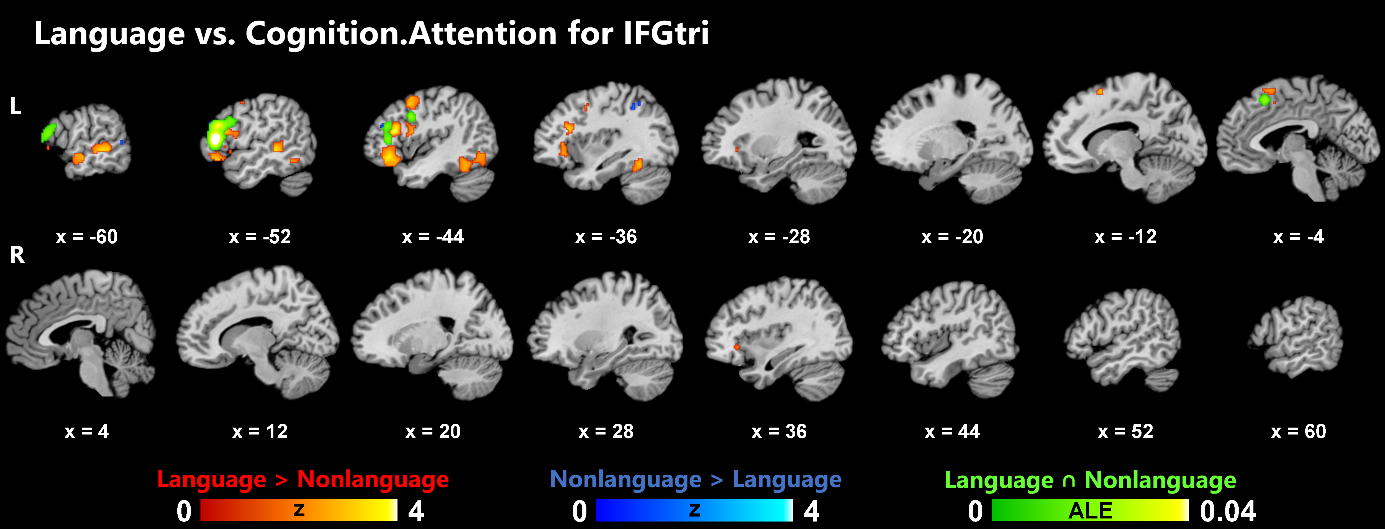


Figure SI1. Contrast and conjunction results of the language and attention (nonlanguage) domains for IFGtri. Color bars indicate *Z* scores for contrast and ALE scores for conjunction analyses.
